# FO‐SPR biosensor calibrated with recombinant extracellular vesicles enables specific and sensitive detection directly in complex matrices

**DOI:** 10.1002/jev2.12059

**Published:** 2021-02-23

**Authors:** Yagmur Yildizhan, Venkata Suresh Vajrala, Edward Geeurickx, Charles Declerck, Nevena Duskunovic, Delphine De Sutter, Sam Noppen, Filip Delport, Dominique Schols, Johannes V. Swinnen, Sven Eyckerman, An Hendrix, Jeroen Lammertyn, Dragana Spasic

**Affiliations:** ^1^ Department of Biosystems Biosensors group, KU Leuven Leuven Belgium; ^2^ Department of Human Structure and Repair Laboratory of Experimental Cancer Research Ghent University Ghent Belgium; ^3^ VIB Center for Medical Biotechnology & Department of Biomolecular Medicine Ghent University Ghent; ^4^ Department of Microbiology Immunology and Transplantation, Laboratory of Virology and Chemotherapy, Rega Institute KU Leuven Leuven Belgium; ^5^ FOx Biosystems Bioville Diepenbeek Belgium; ^6^ Department of Oncology Laboratory of Lipid Metabolism and Cancer KU Leuven Leuven Belgium

**Keywords:** biosensor, complex matrices, extracellular vesicles, fiber optics, surface plasmon resonance

## Abstract

Extracellular vesicles (EVs) have drawn huge attention for diagnosing myriad of diseases, including cancer. However, the EV detection and analyses procedures often lack much desired sample standardization. To address this, we used well‐characterized recombinant EVs (rEVs) for the first time as a biological reference material in developing a fiber optic surface plasmon resonance (FO‐SPR) bioassay. In this context, EV binding on the FO‐SPR probes was achieved only with EV‐specific antibodies (e.g. anti‐CD9 and anti‐CD63) but not with non‐specific anti‐IgG. To increase detection sensitivity, we tested six different combinations of EV‐specific antibodies in a sandwich bioassay. Calibration curves were generated with two most effective combinations (anti‐CD9/^B^anti‐CD81 and anti‐CD63/^B^anti‐CD9), resulting in 10^3^ and 10^4^ times higher sensitivity than the EV concentration in human blood plasma from healthy or cancer patients, respectively. Additionally, by using anti‐CD63/^B^anti‐CD9, we detected rEVs spiked in cell culture medium and HEK293 endogenous EVs in the same matrix without any prior EV purification or enrichment. Lastly, we selectively captured breast cancer cell EVs spiked in blood plasma using anti‐EpCAM antibody on the FO‐SPR surface. The obtained results combined with FO‐SPR real‐time monitoring, fast response time and ease of operation, demonstrate its outstanding potential for EV quantification and analysis.

## 1 INTRODUCTION

Extracellular vesicles (EVs) are nanometre‐sized particles, secreted by both eukaryotic and prokaryotic cells that contain lipids, proteins, nucleic acids and metabolites (Yáñez‐Mó et al., 2015). As per their size and the route of formation, EVs are classified into three main types, exosomes (30–150 nm), microvesicles (100–1000 nm) and apoptotic bodies (100–5000 nm) (Van Niel et al., 2018). EVs actively travel in intercellular matrices, eventually reaching the circulation, and potentially provide a minimally invasive route to access disease specific proteomic and genomic biomarkers (Raghu et al., 2018). In this context, analysis of EVs has drawn huge attention lately for diagnosis, prognosis and therapy of different diseases, such as diabetes (Pardo et al., 2018; Xing et al., 2020), obesity (Pardo et al., 2018), neurodegenerative diseases (Croese & Furlan, 2018), cardiovascular diseases (Khoei et al., 2020) and cancer (Bebelman et al., 2018; Wendler et al., 2017; Xu et al., 2018). However, there are multiple aspects that often introduce an extra level of complexity in studying, characterizing and finally understanding the full potential of EVs, such as: (1) the existence of numerous methods for separating and characterizing EVs, (2) the intrinsic heterogeneity of EV subtypes with varying size (from 40 to >500 nm), molecular patterns, and origin, and (3) the complexity of biofluids (Tkach et al., 2017; Tulkens et al., 2020; Van Deun et al., 2017).

The additional contributing factor to this complexity is the lack of analytical instruments for high quality EV analyses that are at the same time well‐calibrated, user‐friendly and cost‐effective. There are several available methods to perform EV analysis, such as western blot (Kowal et al., 2017), enzyme‐linked immunosorbent assay (ELISA) (Zarovni et al., 2015), flow cytometry (Stoner et al., 2016), nanoparticle tracking analysis (NTA) (Gardiner et al., 2014), transmission electron microscopy (Van der Pol et al., 2014), electrochemistry (Jeong et al., 2016), tunable resistive pulse sensing (TRPS) (Vogel et al., 2016) and surface plasmon resonance (SPR) (Rupert et al., 2014; Zhu et al., 2014). However, many of them have various downsides such as inaccuracy, reliance on complex and/or high‐cost instruments, inability to directly detect EVs in complex matrices or combinations thereof. For instance, flow cytometry is one of the most commonly used techniques for EV analysis (Van der Pol et al., 2014) where particle detection is based on scattering, with silicon or polystyrene beads used as a reference material (Chandler et al., 2011). Nevertheless, flow cytometry often underestimates actual EV concentrations, since the refractive index (RI) of these beads is higher than EVs in general and the EV concentration (particle count) decreases with increase in diameter (Vestad et al., 2017). Likewise, in the case of NTA, which derives the hydrodynamic diameter of EVs based on their Brownian motion, the RI and EV size distribution can affect the measured EV concentration. Moreover, NTA is unable to distinguish membrane‐enclosed EVs from other extracellular particles of similar size. Another frequently used method is SPR technology that offers a unique advantage of real time and label‐free monitoring of EVs binding to the sensor surface. Although remarkable limit of detection (LOD) values of ∼10^7^ to 10^8^ particles/ml have been reported for SPR‐based platforms (Grasso et al., 2015; Hosseinkhani et al., 2017), the most commonly used ones, including commercially available Biacore remain bulky and expensive. Moreover, number of SPR platforms, including the Biacore, are configured with microfluidics, which makes them prone to clogging when using crude samples (Glynn et al., 2008). This configuration also restricts the usage of gold nanoparticles (AuNPs) for further signal amplification and improved sensitivity, a strategy recently proven useful on other non‐microfluidic SPR platform (Liao et al., 2020). Several advanced micro‐technology‐based plasmonic sensing and imaging strategies were reported also to enable selective EV detection along with improved LOD up to ∼10^5^ EV/ml (Im et al., 2014) and multiplex capacity (Raghu et al., 2018; Thakur et al., 2017; Zhu et al., 2014b). However, they are either limited by the operational complexity or expensive fabrication.

In addition to this, majority of the above mentioned technologies share one common drawback, being lack of standardization as they have been developed using heterogeneous EV material. This is because the EV field has been missing well characterized reference materials with similar properties to EVs that could enable reproducible EV analysis. Few materials were reported so far in literature, such as liposomes (Maas et al., 2015) and nanoerythrosomes (NanoE) (Valkonen et al., 2017). However, real matching of their physical and biochemical properties to different EVs, such as size, RI and molecular content, is still under investigation. In addition, robust and user‐friendly preparation of such materials remains challenging. All this emphasizes the need to develop a specific, selective and user‐friendly biosensor for EV analysis, which is pre‐evaluated with a biological EV reference material. This could result in the wanted standardization of EV analysis research and as such significantly advance the field.

In this context, we used in this paper an in‐house made fiber optic surface plasmon resonance (FO‐SPR) platform (commercialized by FOx Biosystems) together with our recently reported recombinant EVs (rEVs) (Geeurickx et al., 2019) to establish a calibrated platform for EV detection and analysis. The FO‐SPR platform can offer multiple advantages to the EV field, such as sensitivity and specificity of prism‐based SPR devices at the fraction of their cost and complexity. Over the years, our team successfully implemented different bioassays on this platform for detecting DNA (Daems et al., 2018; Knez et al., 2015; Peeters et al., 2019), proteins (Lu et al., 2016, 2017) and bacteriophages (Knez et al., 2013). rEVs originate from medium conditioned by HEK293T cells that express the major structural component of HIV‐1 virus particles, the gag polyprotein. Previously, we demonstrated that rEVs exhibit similar physical and biochemical properties to EVs, are near 100% fluorescent and contain non‐human gag‐EGFP mRNA and protein, which allows to track and differentiate them from endogenous EVs using routine technologies (Geeurickx et al., 2019).

Starting from this well‐characterized biological reference material, we first established FO‐SPR bioassay for specific, label‐free rEV detection by optimizing different aspects, including the reproducible functionalization of capture antibodies on the FO‐SPR surface and selection of detection buffer. Subsequently, the sandwich bioassay was built to increase the sensitivity of rEV detection in buffer, followed by selecting the best performing pairs of capture and detection antibodies to generate calibration curves with a series of rEV concentrations in buffer. Lastly, the specificity and robustness of the established sandwich bioassay was probed in complex matrices, by directly detecting rEVs and HEK293 endogenous EVs in cell culture medium supplemented with exosome‐depleted fetal bovine serum (ED‐FBS). Finally, EVs from MCF7 cancer cell line were detected when spiked in 100‐fold diluted blood plasma. In conclusion, we developed a fully integrated FO‐SPR biosensor, optimized its performance using rEVs and validated its use for detection of (endogenous) EVs directly in complex matrices.

## 2 MATERIALS AND METHODS

### 2.1 Reagents and antibodies

All buffer reagents were obtained from Sigma‐Aldrich (Bornem, Belgium), unless stated otherwise. All buffer solutions were made using deionized water purified with the Milli‐Q Plus system (Millipore, Marlborough, MA, USA). Tween 20 was purchased from AppliChem GmbH (Darmstadt, Germany). Ethanol, hydrochloric acid and sodium hydroxide were obtained from VWR (Leuven, Belgium). Superblock buffer, acetone, 1‐ethyl‐3‐(3‐dimethylaminopropyl) carbodiimide (EDC) and N‐hydroxysuccinimide (NHS) were purchased from Thermo Fisher Scientific (Erembodegem, Belgium). COOH SAM was purchased from GERBU Biotechnik GmbH (Heidelberg, Germany). AuNPs, conjugated with goat anti‐biotin antibody, of 40 nm diameter and the optical density (OD) of 10, were provided by BBI Solutions (Cardiff, UK).

In this study, we have used EV‐validated antibodies, which have been previously characterized through various quality control tests, such as western blot, flow cytometry, ELISA, etc. (as detailed on the manufacturer's websites indicated further in the text). This selection ensured the high quality of the antibodies for this study, essential when developing immunoaffinity‐based approach for EV detection. Mouse monoclonal antibodies anti‐CD9 (Cat. no: EX201‐100) and anti‐CD63 (Cat. no: EX204‐100) were purchased from Cell Guidance Systems Ltd (Cambridge, UK). Mouse monoclonal EpCAM specific antibody (anti‐EpCAM, Cat. no: 324202), as well as biotinylated anti‐CD63 (Cat. no: 353018), biotinylated anti‐CD9 (Cat. no: 312110) and biotinylated anti‐CD81 (Cat. no: 349514) antibodies (^B^anti‐CD63, ^B^anti‐CD9 and ^B^anti‐CD81, respectively) were obtained from Biolegend (ImTec Diagnostics, Antwerp, Belgium). Goat anti‐mouse IgG antibody (anti‐IgG) was supplied by Life Technologies (Cat. no: 31430) (Merelbeke, Belgium). All antibody concentrations are indicated in the following sections.

Pooled plasma was obtained from more than 25 healthy donors with a signed informed consent form, recruited at the Laboratory for Thrombosis Research (KU Leuven, Campus Kulak Kortrijk, Belgium). 6 tubes per donor (+/‐ 35 ml blood per donor) were collected using BD vacutainer trisodium citrate tubes (BD 366575, BD, Temse, Belgium). All the tubes were pooled together into 50 ml tubes and centrifuged for 15 min at 2200 rpm. Plasma was collected with a sterile pipette (612‐1685, VWR, Leuven, Belgium) and combined in a sterile container on ice. When all blood has been processed, the pooled samples were placed in the warm water bath at 37°C for 7 min. 10 ml aliquots in 15 ml tubes were prepared from the mixture and stored in boxes at ‐80°C.

### 2.2 Separation and storage of rEVs and EVs

rEVs and MCF7 EVs were separated from the culture medium of HEK293T cells and MCF7 cells, respectively, as previously described (Geeurickx et al., 2019). HEK293 endogenous EVs were isolated from the culture medium of HEK293 cells. HEK293 cells were cultured in Dulbecco's modified Eagle's medium (DMEM; Thermo Fisher Scientific, Carlsbad, CA, USA) supplemented with 0.01 M Hepes (Thermo Fisher Scientific), 1 mM sodium pyruvate (Thermo Fisher Scientific) and 10% heat‐inactivated ED‐FBS (System Biosciences, Palo Alto, CA, USA). Cells were seeded at 10.000 cells/cm² and cultured for 72 h at 37°C in a humidified 5% CO_2_ incubator. Cells were stained with trypan blue to assess cell viability and counted with the Luna II automated cell counter (Westburg, Leusden, The Netherlands). At the moment of supernatant collection, 90.000 cells/cm² were counted with a viability of 88%. The supernatant was collected and sequentially centrifuged for 6 min at 300 × *g* and for 10 min at 3000 × *g* to remove, respectively, dead cells and cell debris. Finally, the supernatant was filtered using a 0.2 μm filter (Thermo Fisher Scientific) and acidified to pH 6 for FO‐SPR measurements.

Aliquots of rEVs, HEK293 endogenous EVs and MCF7 EVs were stored at ‐80°C until further use and thawed carefully on ice just before performing the analysis. We have submitted all relevant data of our experiments to the EV‐TRACK knowledgebase (EV‐TRACK ID: EV200047) (Van Deun et al., 2017).

### 2.3 Characterization of EVs using NTA

The NTA (Jeong et al., 2016) was performed using EV stock solution diluted in phosphate buffer saline (PBS) buffer to a final volume of 800 μl. The concentration and particle size distribution of EV samples were determined using a NanoSight LM10 (Malvern Instruments, Worcestershire, UK) configured with a 405 nm laser. An sCMOS camera with varying shutter lengths was used for recording. The detection threshold was set between 4 to 5, and 6 videos of 30 s were taken. Calculations were performed with the NanoSight NTA analytical software (version 2.3, Nanosight Ltd, Wiltshire, UK). Particle size distribution plots of rEVs, HEK293 endogenous EVs and MCF7 EVs are presented in the Supplementary information (Figure S1).

### 2.4 FO‐SPR biosensor and manufacturing of FO‐SPR probes

**FIGURE 1:**
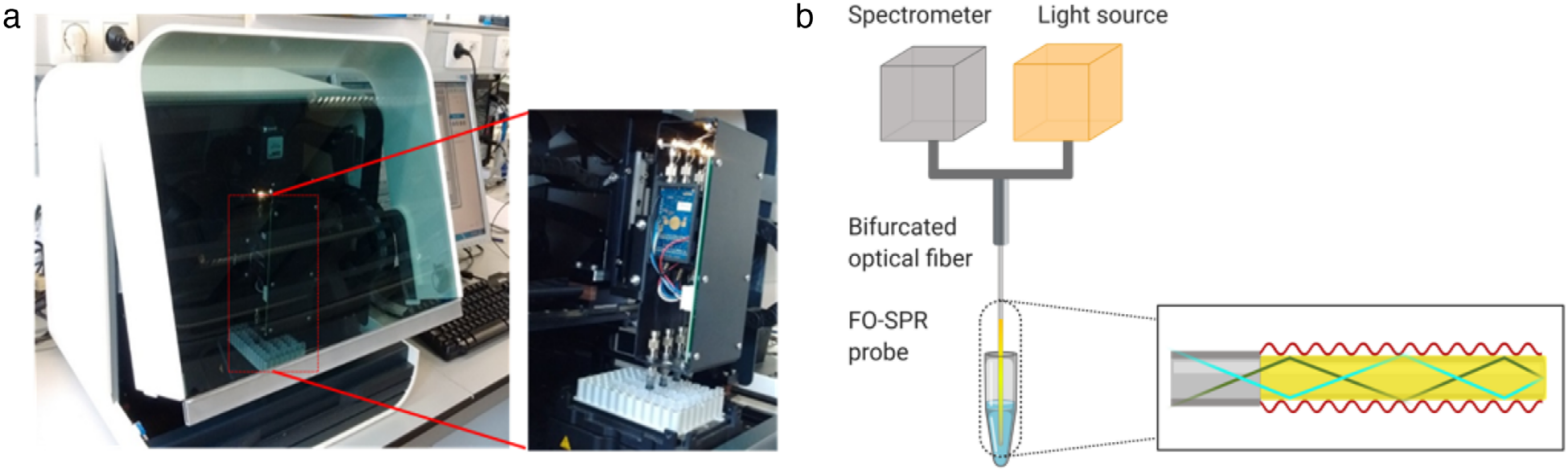
(a) Picture of the FO‐SPR platform (White FOx 1.0, commercialized by FOx Biosystems) used for EV analysis in this paper. (b) The schematic of FO‐SPR working principle (created with Biorender.com)

SPR is the gold standard technology for real‐time analysis of biomolecular interactions. An analytical benchtop FO‐SPR biosensor (Figure 1a) has been introduced by our group and commercialized by FOx Biosystems (Belgium) as an alternative to the traditional prism‐based SPR systems, like Biacore (Qu et al., 2020).

The FO‐SPR platform allows simultaneous and completely automated measuring of four samples through four separate FO‐SPR probes. These probes are connected through a bifurcated fiber to 4 different broad‐spectrum white light LED sources and spectrophotometers (USB4000, Ocean Insight, FL, USA). The bifurcated fiber enables white light to travel through the sensor tip, covered with a 50 nm gold layer, where it is reflected back to the spectrophotometer. An SPR is generated in this gold layer where the light interacts with the surface of the optical fiber. A biomolecular binding event or any alteration in the refractive index on the outside of the gold layer interrupts surface plasmons, changing the resonance conditions and shifts the resonance wavelength. This working principle allows many biomolecular interactions to be monitored in real time.

The optical assembly of the device is protected and covered by a black container. Before starting the experiment, the sequence of dipping the FO‐SPR probes into different solutions was loaded into the custom‐made software, while the PCR tubes (VWR international, Belgium), filled with different buffers and reagents, were placed in the system tube holder. Next, the functionalized probes were attached to the FO‐SPR sensor and parallel measurements were conducted. User‐friendly software was used to control the automated robot system, enabling a flexible movement of the FO‐SPR probes and recording the obtained data.

The FO‐SPR probes were prepared as previously described (Arghir et al., 2015; Pollet et al., 2009). Briefly, FO‐SPR probe was cut starting from a multimode optical fiber (TEQS, Thorlabs, Munich, Germany) with a core diameter of 400 μm and a length of 4.3 cm. The fiber cleavage was achieved by using an LDC‐400 device (Vytran, UK). FO‐SPR probe sensitive zone was prepared by removing approximately 0.6 cm of the jacket from one end of the fiber (using FO stripper) and by subsequently removing the cladding with an acetone‐soaked dust free tissue. Next, FO‐SPR probe was cleaned with ethanol and a thin layer (∼50 nm) of gold was sputtered using a sputter coater (Quorum Q150T ES, Quorum Technologies, East Sussex, UK). Gold coated FO‐SPR probes were functionalized at 4°C in a 0.1 mM ethanol/COOH SAM (volume ratio of 9:1) for 2 days and used immediately afterwards in the experiments. Just before the experiment, the probes were rinsed with ethanol to remove any unbound material.

### 2.5 Surface functionalization of the FO‐SPR probes with antibodies

**FIGURE 2:**
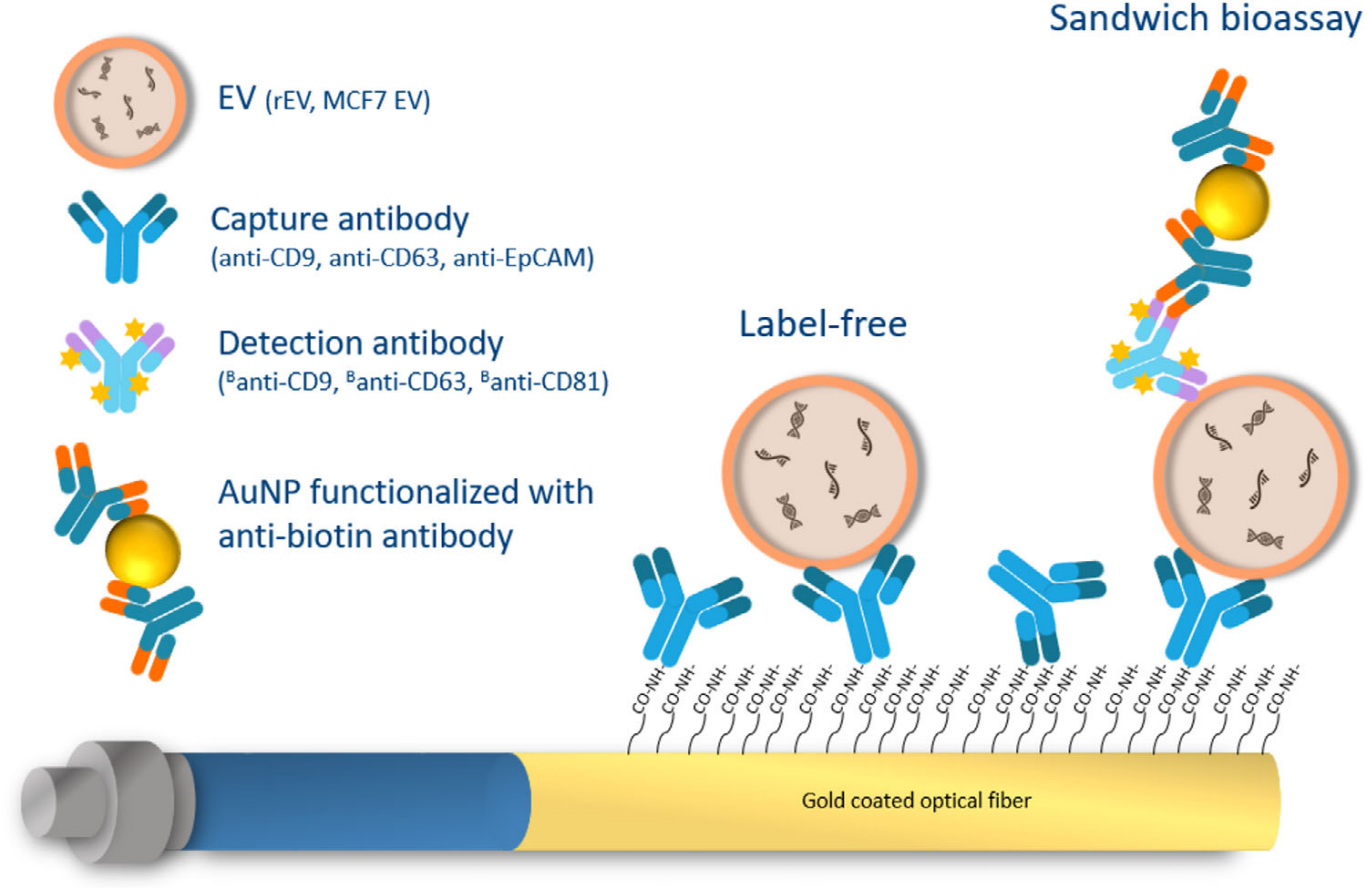
A schematic overview of the EV detection bioassay. Carboxylic acid self‐assembling monolayer (COOH SAM) was first formed on the surface of a gold coated FO‐SPR probe. Capture antibodies (anti‐CD9, anti‐CD63 or anti‐EpCAM) were covalently immobilized on the surface through activated COOH groups. EVs were bound to the capture antibodies in a label‐free manner. The signal was then amplified by using biotinylated detection antibodies (^B^anti‐CD9, ^B^anti‐CD63 or ^B^anti‐CD81) and AuNPs functionalized with anti‐biotin antibody. Stars on detection antibodies depict biotin

**FIGURE 3:**
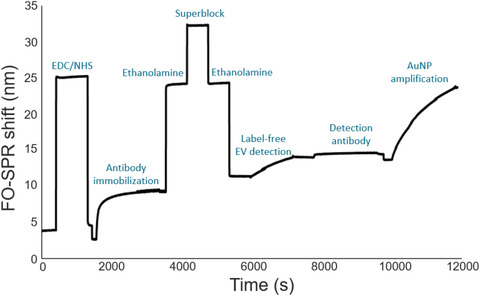
FO‐SPR sensorgram depicting each step of the bioassay, as detailed in Section 2.5

As shown in Figure 2 and with the real‐time sensorgram in Figure 3, COOH SAM functionalized FO‐SPR probes were first immersed in a freshly made mixture of 0.4 M EDC and 0.1 M NHS, dissolved in 50 mM MES buffer at pH 6, for 15 min to activate COOH groups of the SAM. Next, EV specific capture monoclonal antibodies (anti‐CD9, anti‐CD63 and anti‐EpCAM) were initially diluted in 3 different buffers (50 mM MES at pH 6 and 10 mM sodium acetate at pH 5.2 or pH 5.6) at a concentration of 20 μg/ml and were immobilized on the FOR‐SPR probe with gentle shaking at 200 rpm, for 30 min. During this step, the antibodies were bound covalently to the activated COOH groups. Subsequently, the FO‐SPR probes were immersed sequentially in blocking buffers: 1 M ethanolamine at pH 8, Superblock and again in 1 M ethanolamine to minimize the non‐specific binding. Based on the obtained results (see further), 10 mM sodium acetate at pH 5.6 was selected to functionalize the FO‐SPR surface with anti‐CD9, anti‐CD63 and anti‐EpCAM capture antibodies in all the subsequent experiments.

### 2.6 Label‐free and sandwich FO‐SPR bioassay for detection of rEVs in buffer

The FO‐SPR probes, functionalized as described in the Section 2.5, were subsequently used for rEV detection in buffer. 50 mM MES detection buffer with several pH values (5, 5.5, 6 and 6.5) and PBS pH 7.4, all supplemented with 0.01% BSA and 0.01% Tween 20 were tested initially, resulting in 50 mM MES pH 6 as the most optimal for rEV detection (see further), which was then used in all the subsequent experiments. Each FO‐SPR probe was immersed in the detection buffer to obtain a baseline signal. Next, the FO‐SPR probe was introduced to the detection buffer containing rEVs at different concentrations, ranging from 0 (negative control) to 2 × 10^9^ particles/ml (with 2‐fold dilution), for 20 min to record the real‐time, label‐free binding of rEVs. In order to achieve signal amplification, the FO‐SPR probe was subsequently immersed in the same detection buffer with 10 μg/ml concentration of detection antibodies (^B^anti‐CD63, ^B^anti‐CD9 or ^B^anti‐CD81) for 30 min at 200 rpm. Afterwards, the FO‐SPR probe was re‐introduced into the detection buffer, followed by immersion into the PBS with 0.5% of BSA to obtain a baseline signal for the next step with goat anti‐biotin conjugated AuNPs. These AuNPs were prepared, prior using in the FO‐SPR bioassay, by centrifugation at 5000 rpm for 30 min at 4°C. After removing the supernatant, AuNPs were re‐suspended in PBS with 0.5% of BSA with 1:10 dilution ratio to decrease the OD from 10 to 1. FO‐SPR probes were immersed into 150 μl solution of AuNPs for 1 h without any shaking. All the steps of rEV detection bioassay were performed at room temperature and are depicted with the sensorgram in Figure 3.

### 2.7 Visualization of rEVs bound onto the FO‐SPR probe using fluorescence microscopy

The rEVs were first captured by anti‐CD9 antibody immobilized on the FO‐SPR probe at a concentration of 5 × 10^8^ particles/ml. This was performed in the detection buffer (50 mM MES pH 6, 0.01% BSA, 0.01% Tween 20), followed by carefully mounting the FO‐SPR probe on a coverslip, containing the same buffer. Subsequently, the visualization was done on the Nikon Ti‐Eclipse inverted microscope (Nikon, Japan) based on the intrinsic GFP fluorescence of rEVs (Geeurickx et al., 2019). FO‐SPR probes functionalized with anti‐IgG antibody were used in this experiment as negative control.

### 2.8 FO‐SPR bioassay for detecting EVs directly in cell culture medium and plasma

The FO‐SPR probes, functionalized as described in the Section 2.5, were also used for detecting: (1) rEVs at concentration of 2 × 10^9^ particles/ml and (2) HEK293 endogenous EVs at concentration of 6.8 × 10^8^ particles/ml both directly in DMEM cell medium supplemented with 10% ED‐FBS and with anti‐CD63 as capture antibody, as well as (3) MCF7 EVs spiked in 100‐fold diluted plasma samples at concentration of 2 × 10^9^ particles/ml with anti‐EpCAM as capture antibody. The bioassay was performed in the same way as described in Section 2.6, with the ^B^anti‐CD9 as detection antibody for rEVs and HEK293 endogenous EVs or ^B^anti‐CD63 as detection antibody for detecting MCF7 EVs.

### 2.9 Data analysis

The obtained data were recorded by using a custom‐built software developed by FOx Biosystems Ltd and further processed in Excel. The noise (i.e. FO‐SPR signal obtained from blank measurements) was subtracted from all the FO‐SPR shifts obtained for different EV concentrations prior building calibration curves. Calibration curves were fitted throughout the entire measured concentration range by applying non‐linear curve fitting using specific binding equation from GraphPad Prism software: $Y\;=\frac{A*X}{B+X}\;$. The measured LOD was determined as the sum of the respective blank signal and 3 times the standard deviation of the blank signal. The signal to noise ratios (SNR) was always calculated by dividing specific with the background signal, the latter obtained for samples without EVs (0 particles/ml).

## 3 RESULTS AND DISCUSSION

### 3.1 FO‐SPR surface functionalization with EV specific antibodies

**FIGURE 4:**
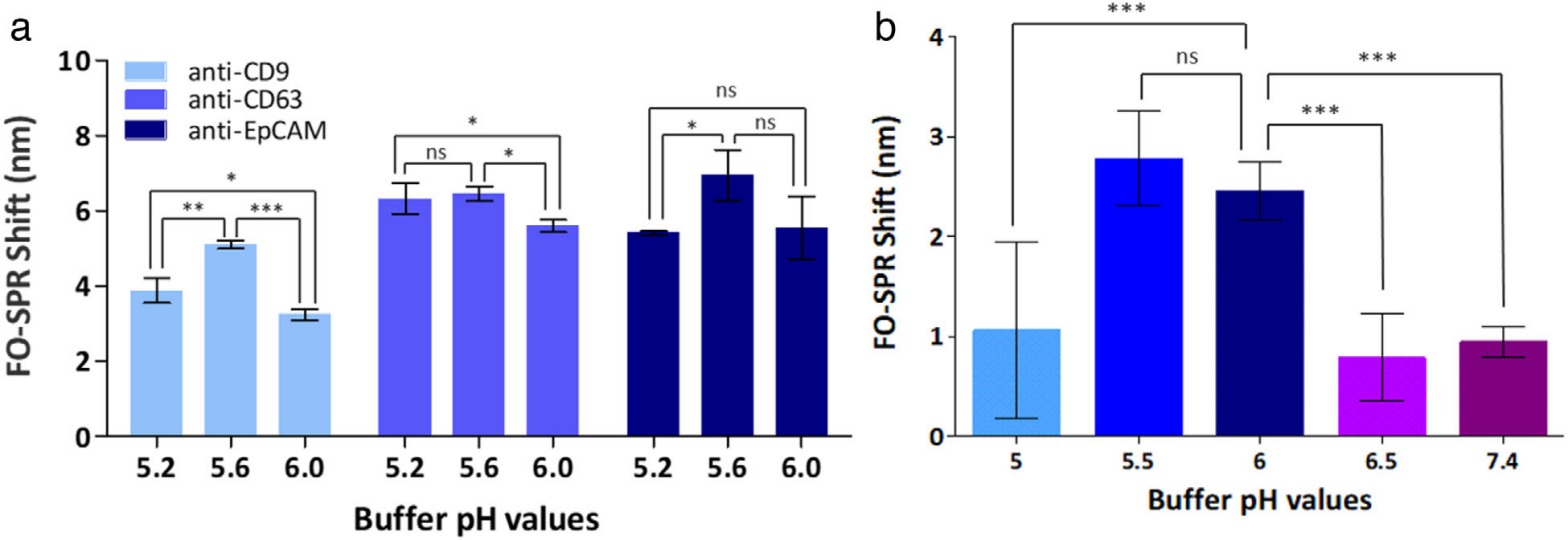
(a) Bar graph representing the FO‐SPR shifts when immobilizing three different antibodies (anti‐CD9, anti‐CD63 and anti‐EpCAM) using three different immobilization buffers (10 mM sodium acetate buffer pH 5.2 or 5.6 and 50 mM MES buffer pH 6.0). Error bars represent the standard deviation (n = 3). (b) Bar graph representing the FO‐SPR shifts when testing rEV binding to the sensor surface, functionalized with anti‐CD9, at different pH values (ranging from 5.0 to 6.5) of 50 mM MES buffer and in PBS pH 7.4. Error bars represent the standard deviation (n = at least 2). The statistical difference between the groups was assessed by 1way ANOVA with Tukey's multiple comparison test, where *, ** and *** represents *P* < 0.05 and ns represents no significant difference. The statistical analysis were performed using GraphPad prism software (GraphPad Software, CA, US)

Experiments were first carried out to establish conditions for the robust immobilization of EV‐specific antibodies on the biosensor surface. We selected 2 antibodies specific to the generic EV biomarkers, anti‐CD9 and anti‐CD63 (Rosa‐Fernandes et al., 2017) and 1 antibody for the cancer specific EV biomarker, anti‐EpCAM (Osta et al., 2004). As we showed previously (Lu et al., 2016), the efficiency of antibody immobilization depends on antibody concentration, immobilization time, ionic strength and buffer pH value. Here, we selected 20 μg/ml as the antibody concentration that results in surface saturation for most of the antibodies on the FO‐SPR surface (Lu et al., 2016, 2017), while testing several pH values since the isoelectric points (pI) of these selected antibodies were unknown. For maximizing the immobilization efficiency, we used buffers with lower ionic strength, i.e. 10 mM sodium acetate buffer (pH 5.2 and 5.6) and 50 mM MES buffer (pH 6.0). Based on the results from Figure 4a, we selected 10 mM sodium acetate buffer pH 5.6 as the most optimal for immobilizing all 3 capture antibodies, since (1) it resulted in the highest FO‐SPR shift for anti‐CD9 antibody whereas (2) for the other 2 antibodies this condition was better than at least one of the other 2 tested buffers.

### 3.2 Optimizing conditions for specific label‐free detection of rEVs in buffer

For establishing robust and specific rEV binding to the FO‐SPR biosensor, we first tested 5 detection buffers: 50 mM MES buffer at pH values ranging from 5.0 to 6.5 and 10 mM PBS pH 7.4, all supplemented with 0.01% BSA and 0.01% Tween 20. FO‐SPR probes were functionalized with anti‐CD9 for detecting 3 × 10^9^ particles/ml of rEVs (determined based on the NTA). Among all tested conditions, the highest FO‐SPR shift values were obtained at pH 5.5 and 6 with no statistically significant differences between them (Figure 4b). Because of smaller variability, 50 mM MES buffer pH 6 was selected as detection buffer for all subsequent experiments.

**FIGURE 5:**
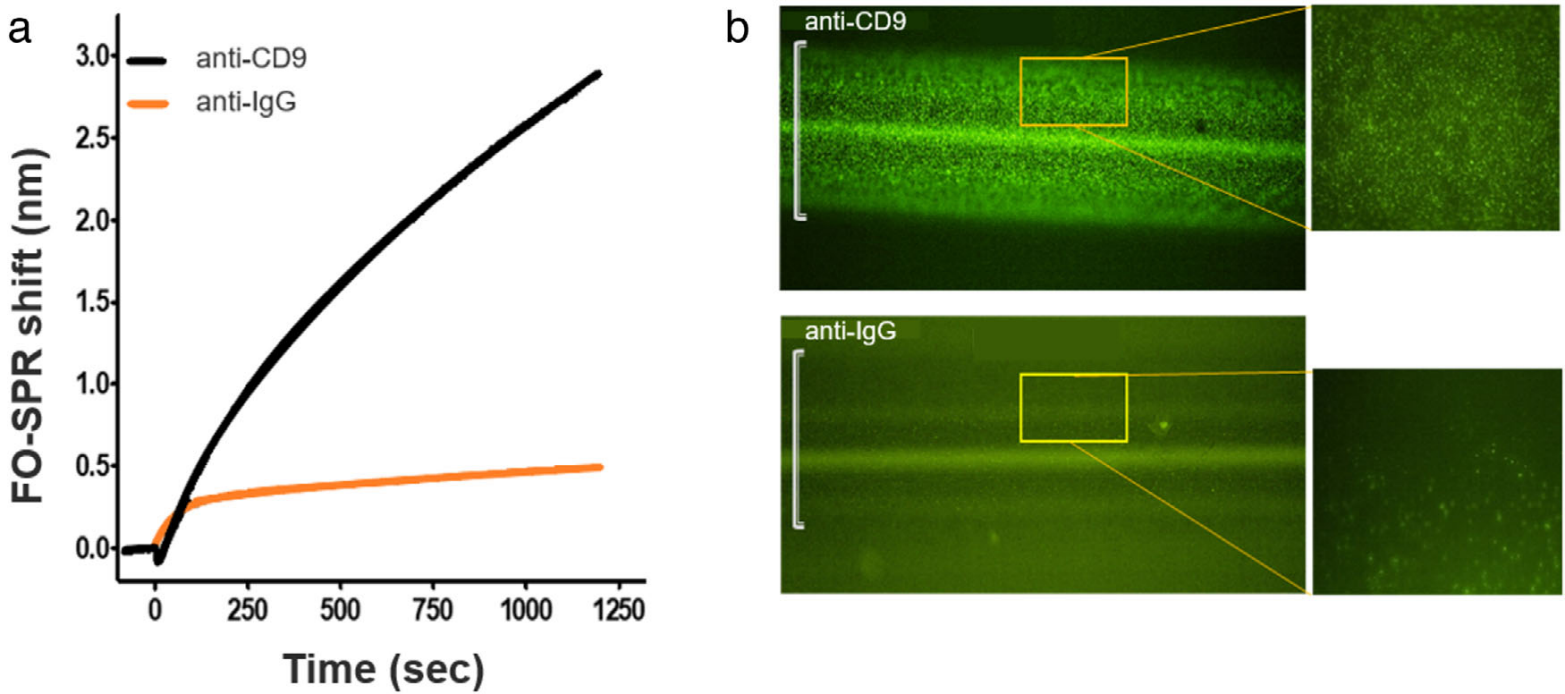
(a) FO‐SPR sensorgram depicting the label‐free detection of rEVs (3 × 10^9^ particles/ml) using EV specific and non‐specific antibodies (i.e. anti‐CD9 and anti‐IgG, respectively). FO‐SPR shift (nm) changes were plotted as a function of time in seconds. (b) Microscopic verification of captured rEVs on the FO‐SPR sensor surface functionalized with anti‐CD9 or anti‐IgG antibodies. The images were taken using an inverted fluorescence microscope at 10x and 40x (inset) magnification. Green bright spots on the FO‐SPR surface represent the intrinsic GFP fluorescence of rEVs (ex‐488 nm/ em‐507 nm) whereas the white brackets on the microscopy images depict the width of the FO‐SPR probe core (400 μm)

It has been shown already for EVs that their stability and ability to properly deliver signals depends on their zeta potential as well as on the pH and the ionic strength of the surrounding biological fluid (Beit‐Yannai et al., 2018; Yang et al., 2018). This suggests that the observed pH sensitive EV binding to the FO‐SPR surface might be also due to the surface charge effects. However, pH‐induced attraction to the sensor surface could increase both specific and non‐specific EV binding. To test this, the FO‐SPR probes functionalized with anti‐CD9 were compared with probes functionalized with anti‐IgG (negative control) in the same detection buffer. The bioassay sensorgram showed that rEV binding signal was at least 6 times higher for anti‐CD9 than for the negative control, indicating a specific rEV binding to the FO‐SPR probe functionalized with rEV specific antibody (Figure 5a). This difference was further confirmed under the fluorescence microscope thanks to the intrinsic GFP fluorescence of rEVs, (Figure 5b), which supported the establishment of a specific FO‐SPR bioassay for label‐free rEV detection.

### 3.3 Development of FO‐SPR sandwich bioassay for detecting rEVs in buffer

To enable rEV detection at lower concentrations, we further developed a FO‐SPR sandwich bioassay. Starting from the FO‐SPR probes functionalized with anti‐CD9 or anti‐CD63 antibodies (as described in the previous sections), the sandwich bioassay was established using biotinylated detection antibody followed by AuNPs functionalized with anti‐biotin antibody (Figure 2). As previously shown by our group, AuNPs in FO‐SPR bioassays assure significant signal amplification, and as such more sensitive detection of targets, thanks to the additional mass of AuNPs and plasmon coupling effect between gold surfaces (Lu et al., 2016, 2017; Qu et al., 2020). Here, we have used for the first time an innovative 2‐step signal amplification approach, rather than the well‐established direct immobilization of detection antibodies on AuNPs. This new strategy proved to be essential to match (1) the requirements for low pH 6 of the EV detection buffer (Figure 4b) while (2) enabling to use PBS pH 7.4 for the final step with AuNPs, thus avoiding their clustering at lower pH values.

**FIGURE 6:**
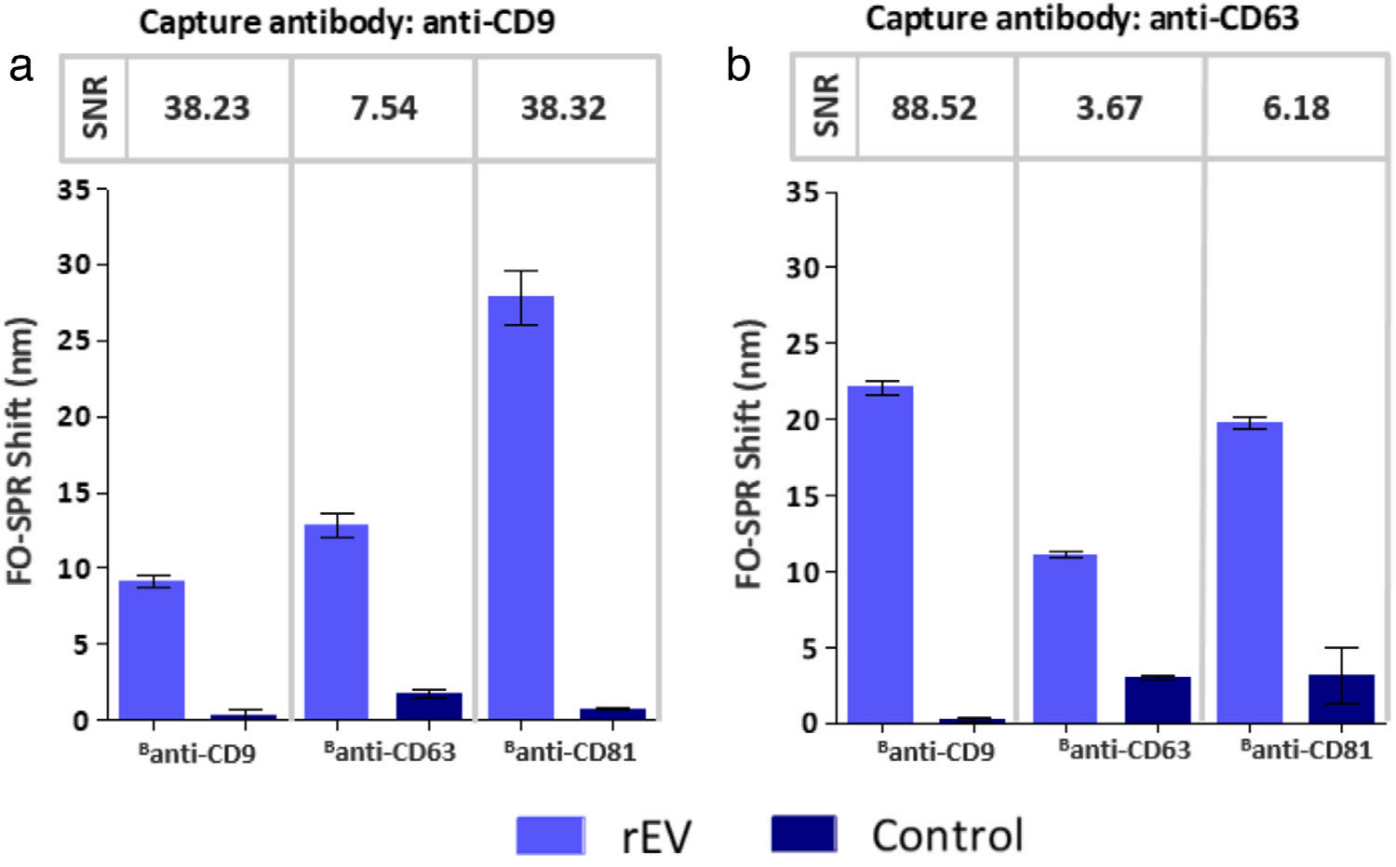
FO‐SPR sandwich bioassay with different antibody combinations for detecting rEVs in buffer. Bar graphs represent the FO‐SPR shifts obtained by combining capture antibodies (a) anti‐CD9 and (b) anti‐CD63 with different detection antibodies (^B^anti‐CD9, ^B^anti‐CD63, ^B^anti‐CD81) for detecting rEVs at 2 × 10^9^ particles/ml concentration. Controls were performed for each antibody combination without rEVs. Respective SNR values are indicated on top of the bars. Error bars represent the standard deviation (n = 2)

Because EVs have multiple copies of the same protein in their membranes, FO‐SPR probes functionalized with anti‐CD9 or anti‐CD63 capture antibodies were tested with both ^B^anti‐CD9 and ^B^anti‐CD63 detection antibodies. Next to this, we also included ^B^anti‐CD81 detection antibody against another commonly reported EV biomarker, that is, CD81. All these combinations have been probed in buffer with fixed concentration of rEVs (2 × 10^9^ particles/ml) and detection antibodies at 10 μg/ml concentration, followed by AuNPs. Figure 6 depicts both the specific (2 × 10^9^ particles/ml) and background signal of the negative controls (0 particles/ml), with the SNR calculated for each antibody combination as explained in Section 2.9. Based on these results, it seemed that all antibody combinations gave respectable SPR shifts of at least 10 nm when rEVs were present in the sample, which were all significantly different from the controls. Although this suggested that all tested antibody combinations can be used for specifically detecting rEVs on the FO‐SPR platform, some were evidently performing better than the others. The observed differences can be explained with the AuNP step itself since FO‐SPR shift for the label‐free detection was very similar for both anti‐CD9 and anti‐CD63 capture antibodies (data not shown).

Nevertheless, the highest SPR shift did not necessarily result in the highest SNR values, pointing to the higher background signal for some combinations (Figure 6a and b). Interestingly, the same combinations of antibodies could also result in hugely different SNR values only if placed in different positions in the established bioassay (i.e. anti‐CD9/^B^anti‐CD63 and anti‐CD63/^B^anti‐CD9 gave 7.54 and 88.52 SNR values, respectively). This can be explained by a combined effect of slightly higher specific signal and lower control signal for the latter, collectively improving the SNR more than 11 fold. Similar effect on the final calculated SNR was also observed for the 2 combinations employing ^B^anti‐CD81 as detection antibody. Although specific signal was at least 20 nm for both conditions, high background signal when using anti‐CD63 as capture antibody resulted in 6‐fold lower SNR.

Because we further wanted to explore the limits of sensitivity for developed sandwich bioassay, we selected the 2 best combinations of antibodies based on the obtained SNR values, being anti‐CD9/ ^B^anti‐CD81 and anti‐CD63/ ^B^anti‐CD9.

### 3.4 Quantification of rEVs in buffer using FO‐SPR sandwich bioassay

**FIGURE 7:**
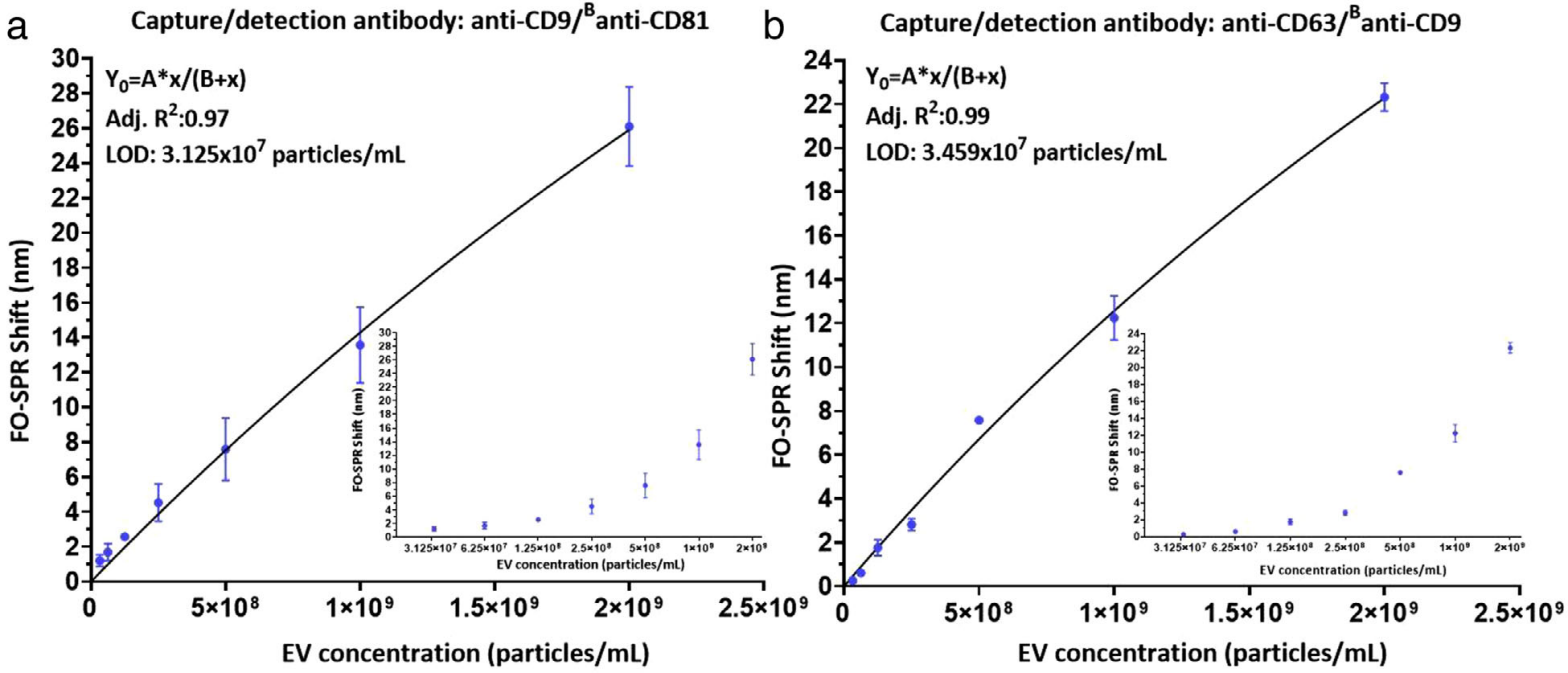
FO‐SPR based detection of a series of rEV concentrations in the detection buffer (50 mM MES pH6, 0.01% BSA, 0.01% Tween 20) when using: (a) anti‐CD9/ ^B^anti‐CD81 and (b) anti‐CD63/^B^anti‐CD9 antibody combinations. FO‐SPR shifts (nm) were plotted as a function of rEV concentrations (particles/ml). Non‐linear curve fittings were performed by prism software using specific binding equation: $Y=\frac{A*x}{B+x}$. Inset graphs are included to demonstrate the obtained FO‐SPR shifts in function of EV concentrations on log2 scale for better signal resolution in the lower concentration range. Error bars represent standard deviations (n = 3).

The 2 best combinations of antibodies from Figure 6 (i.e. anti‐CD9/^B^anti‐CD81 and anti‐CD63/^B^anti‐CD9) were used to build the calibration curves in buffer with a series of rEV concentrations, ranging from 3.125 × 10^7^ to 2 × 10^9^ particles/ml (including also a negative control without any EVs, 0 particles/ml). The obtained average FO‐SPR shifts (n = 3) were plotted as a function of rEV concentrations and the calibration curves were fitted as detailed in Section 2.9 (Figure 7). The dose‐response calibration curves showed that FO‐SPR biosensor detected rEVs over the entire tested concentration range for both antibody combinations with measured LOD values (as explained in Section 2.9) of 3.125 × 10^7^and 3.459 × 10^7^ particles/ml for anti‐CD9/^B^anti‐CD81 and anti‐CD63/^B^anti‐CD9 antibody combinations, respectively. Notably, the obtained LODs were 10^3^ times lower than the expected physiological concentration of EVs in healthy human plasma and 10^4^ times lower than EV concentration in plasma of cancer patients (Geeurickx et al., 2019), demonstrating therefore a huge potential of the established FO‐SPR bioassay for sensitive EV analysis.

### 3.5 Establishing FO‐SPR sandwich bioassay for detection of EVs in complex matrices

**FIGURE 8:**
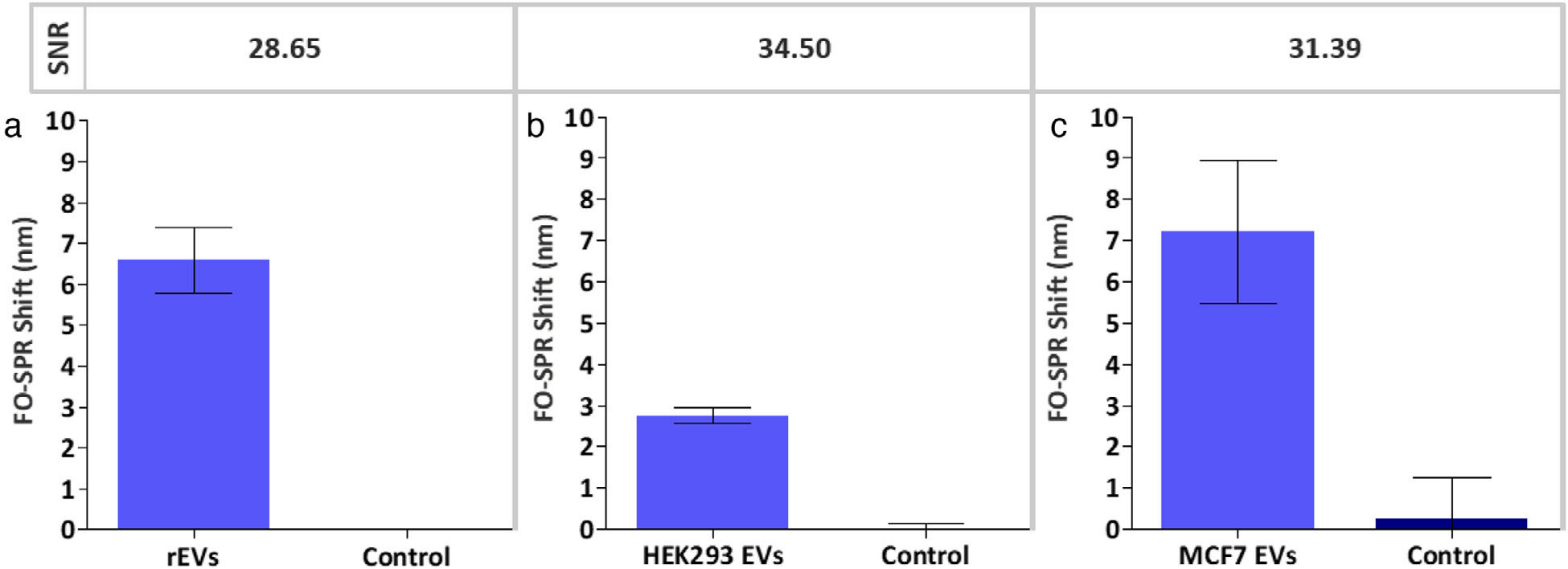
Bar graphs representing the FO‐SPR shifts obtained when detecting (a) rEVs spiked at 2 × 10^9^ particles/ml in DMEM cell culture medium supplemented with 10% ED‐FBS and (b) endogenous HEK293 EVs (6.8 × 10^8^ particles/ml) in DMEM cell culture medium supplemented with 10% ED‐FBS, using for both conditions anti‐CD63 as capture and ^B^anti‐CD9 as detection antibody as well as (c) MCF7 EVs spiked at 2 × 10^9^ particles/ml in 100‐fold diluted blood plasma, using anti‐EpCAM as capture and ^B^anti‐CD63 as detection antibody. Controls represent DMEM cell culture medium supplemented with 10% ED‐FBS for A and B whereas control for C is the same blood plasma without spiked MCF7 EVs. Error bars represent standard deviations (n = 2)

Although the established sandwich bioassay demonstrated a remarkable sensitivity in buffer, we wanted to further exploit its potential towards the detection of EVs in complex matrices. Therefore, anti‐CD63/^B^anti‐CD9 combination was employed for direct detection of rEVs (2 × 10^9^ particles/ml) spiked in DMEM cell culture medium supplemented with 10% ED‐FBS. The SNR value (28.65) in Figure 8a proved specific rEV detection even in a complex matrix. Importantly, the non‐specific signal in the absence of any rEVs (control sample) was completely absent. These results revealed for the first time that the FO‐SPR technology was well‐suited to perform direct measurements in the cell culture medium, even in the presence of FBS, which was perfectly in line with our previous studies on the compatibility of this technology with other diverse complex matrices, such as whole blood, plasma, serum, etc. (Lu et al., 2016, 2017; Qu et al., 2020). As such, we proved yet again the robustness of the FO‐SPR bioassays, which significantly broadens the potential applications of FO‐SPR biosensor in the context of EV detection and analysis.

To further challenge the capacity of our established anti‐CD63/^B^anti‐CD9 bioassay, we tried to detect genuine EVs, rather than just the standardized rEV model system. In this context, we first performed bioassay directly in HEK293 cell culture supernatant, i.e. DMEM medium supplemented with 10% ED‐FBS, which contained 6.8 × 10^8^ particles/ml of endogenous EVs, as determined by NTA (Figure S1). Notably, the SNR value (34.50) depicted in Figure 8b demonstrated a successful detection of these EVs without any prior purification or enrichment of EVs, further emphasizing the specificity and robustness of the established FO‐SPR bioassay. Similarly as in Figure 8a, the non‐specific signal in the control sample was negligible.

Lastly, we tested the potential of using FO‐SPR sandwich bioassay for detecting EVs spiked in blood plasma samples. To do so, we first identified an EV model system that expresses a biomarker not commonly found in EVs originating from healthy cells, thereby assuring lower background signal from all the other EVs normally present in plasma samples of healthy donors. In this context, we used EVs from the breast‐cancer MCF7 cell line, previously shown to express tumor‐specific EpCAM biomarker, and matching anti‐EpCAM antibody to specifically capture these EVs once spiked in blood plasma. Following the established protocols from the previous sections, anti‐EpCAM antibody was immobilized on the sensor surface whereas ^B^anti‐CD63 was used as the best performant detection antibody (Figure S2, Supplementary information) together with AuNPs to build a sandwich bioassay. Remarkably, the obtained SNR value (31.39) as well as the actual measured FO‐SPR shifts (Figure 8c) for a defined concentration of MCF7 EVs (2 × 10^9^ particles/ml) revealed their successful detection directly in 100‐fold diluted blood plasma. It is important to note that the obtained SNR value for the MCF7 EVs cannot be compared to the other 2 tested EVs because of (1) completely different bioassay settings, such as employment of different capture/detection antibodies and distinct sample matrices, and (2) different protein expression levels among those EVs, which can also have impact on the final avidity. Nevertheless, these results demonstrated an excellent performance of FO‐SPR in blood plasma as long as relying on cancer‐specific biomarker for EV capture, thereby revealing great potential of this technology for the future work on quantification and molecular profiling of cancer‐derived EVs.

## 4 CONCLUSION

In this study, we reported the development of FO‐SPR biosensor for specific and sensitive detection of EVs directly in various complex matrices, including cell culture medium and blood plasma.

To achieve this, we first established label‐free FO‐SPR bioassay for specific EV detection using rEV as a biological reference material (Geeurickx et al., 2019). Different aspects of the bioassay were optimized, resulting in selection of the best (1) buffer for reproducible immobilization of different capture antibodies on FO‐SPR sensor surface (10 mM sodium acetate buffer pH 5.6) and (2) detection buffer for specific EV detection (50 mM MES buffer pH 6). This specificity was further supported by an SPR and GFP‐mediated microscopy signal measured only when using EV specific antibody (e.g. anti‐CD9) on the FO‐SPR surface rather than a non‐specific anti‐IgG antibody.

To further increase the sensitivity of the label‐free bioassay, we built a FO‐SPR sandwich bioassay in buffer spiked with rEVs (2 × 10^9^ particles/ml) by using 6 different combinations of capture (anti‐CD9 and anti‐CD63) and detection (^B^anti‐CD9, ^B^anti‐CD63 and ^B^anti‐CD81) EV‐specific antibodies. Importantly, we employed for the first time 2‐step signal amplification strategy on the FO‐SPR device by utilizing biotinylated detection antibody and AuNPs functionalized with anti‐biotin antibody (rather than traditional AuNPs functionalized directly with detection antibody). This approach proved to be not only successful for generating significant SPR shifts (reaching close to 28 nm for certain conditions), but also essential for performing EV detection at required low pH 6, while preserving AuNP step in PBS buffer at pH 7.4. The 2 best capture‐detection antibody combinations (anti‐CD9/^B^anti‐CD81 and anti‐CD63/^B^anti‐CD9) were used to generate calibration curves with a series of rEV concentrations in buffer, resulting respectively in the LOD values of 3.125 × 10^7^ and 3.459 × 10^7^ particles/ml. Remarkably, these LODs were 10^3^ times lower than the expected physiological concentration of EVs in human plasma and 10^4^ times lower than EV concentration in plasma of cancer patients (Geeurickx et al., 2019). The achieved sensitivity demonstrates not only huge potential of the established FO‐SPR technology for EV detection and analysis in general, but might particularly be of importance when aiming to detect different subpopulations of EVs by using specific biomarkers (usually present at much lower concentrations), rather than just the total number of EVs in patients’ samples.

Lastly, the developed sandwich bioassay was probed for its robustness and specificity by detecting 3 different types of EVs in 2 different complex matrices. Here, we reported successful detection of rEVs in cell culture medium supplemented with ED‐FBS but also of endogenous EVs originating from HEK293 cells directly in medium used for growing the cells without any prior EV purification or enrichment. Finally, we have demonstrated outstanding potential of the established FO‐SPR biosensor by detecting MCF7 breast cancer cell EVs spiked in 100‐fold diluted blood plasma samples. The specificity of the bioassay was achieved through immobilization of anti‐EpCAM antibody on the FO‐SPR surface, thereby enabling selective capture of MCF7 EVs expressing this biomarker among all the other EVs originating from healthy cells in used plasma samples.

Overall, our study emphasized the importance of using pre‐evaluated standardized material such as rEVs to develop a robust and specific EV detection method by removing uncertainty during early stages of technology development. Moreover, the obtained results, showed (1) remarkable sensitivity of the FO‐SPR device for EV detection and quantification, (2) its possibility to directly measure in complex matrices, such as cell culture medium and blood plasma, and (3) capacity to differentiate cancer‐specific EVs among those originating from healthy cells as long as a cancer‐specific biomarker is present. All this combined with other intrinsic features of the presented benchtop FO‐SPR biosensor platform, such as real‐time monitoring, fast response time, parallel measurements capacity and ease of operation, demonstrate its huge potential for the EV research field.

## CONFLICTS OF INTEREST

Professor Jeroen Lammertyn is a board member of FOx Biosystems, a spin‐off company of KU Leuven commercializing FO‐SPR platforms, next to the principal investigator of the Biosensors group.

## AUTHOR CONTRIBUTIONS

Yagmur Yildizhan: Methodology, Validation, Formal analysis, Investigation, Writing ‐ Original Draft, Visualization. Venkata Suresh Vajrala: Methodology, Validation, Investigation, Writing ‐ Original Draft. Edward Geeurickx: Resources, Writing ‐ Review & Editing. Charles Declerck: Investigation. Nevena Duskunovic: Investigation. Delphine De Sutter: Resources. Sam Noppen: Resources, Writing ‐ Review & Editing. Filip Delport: Writing ‐ Review & Editing. Dominique Schols: Resources, Writing ‐ Review & Editing. Johannes V. Swinnen: Resources, Writing ‐ Review & Editing, Funding acquisition. Sven Eyckerman: Resources, Writing ‐ Review & Editing. An Hendrix: Resources, Writing ‐ Review & Editing, Funding acquisition. Jeroen Lammertyn: Conceptualization, Resources, Writing ‐ Review & Editing, Supervision, Funding acquisition, Project administration. Dragana Spasic: Conceptualization, Methodology, Writing ‐ Original Draft, Supervision, Funding acquisition, Project administration.

## Supporting information

Supporting Information
