# Supplementary material for: FO‐SPR biosensor calibrated with recombinant extracellular vesicles enables specific and sensitive detection directly in complex matrices
Source: J Extracell Vesicles. 2021 Feb 23;10(4):e12059. doi: 10.1002/jev2.12059 (PMC7902528; doi:10.1002/jev2.12059)
Supplement: Supplementary file 1 — Supporting Information [file JEV2-10-e12059-s001.docx]

FO-SPR biosensor calibrated with recombinant extracellular vesicles enables specific and sensitive detection directly in complex matrices

Yagmur Yildizhan^a,¥^, Venkata Suresh Vajrala^a,¥,†^, Edward Geeurickx^b^, Charles Declerck^a^, Nevena Duskunovic^a,§^, Delphine De Sutter^c^, Sam Noppen^d^, Filip Delport^e^, Dominique Schols^d^, Johannes V. Swinnen^f^, Sven Eyckerman^c^, An Hendrix^b^, Jeroen Lammertyn^a,*^, Dragana Spasic^a^

^¥^equal contribution

*corresponding author: jeroen.lammertyn@kuleuven.be, +3216321459, Willem de Croylaan 42, box 2428, B-3001, Leuven, Belgium

^a^Department of Biosystems, Biosensors group, KU Leuven, Leuven, Belgium; ^b^Laboratory of experimental cancer research, Department of Human Structure and Repair, Ghent University, Ghent, Belgium; ^c^VIB Center for Medical Biotechnology & Department of Biomolecular Medicine, Ghent University, Ghent, Belgium; ^d^Department of Microbiology, Immunology and Transplantation, Laboratory of Virology and Chemotherapy, Rega Institute, KU Leuven, Leuven, Belgium; ^e^FOx Biosystems, Bioville, Diepenbeek, Belgium; ^f^Department of Oncology, Laboratory of Lipid Metabolism and Cancer, KU Leuven, Leuven, Belgium; ^†^present address: Laboratoire d'analyse et d'architecture des systèmes, Toulouse, France; ^§^present address: Korea advanced institute of science and technology, Daejeon, Korea.

***
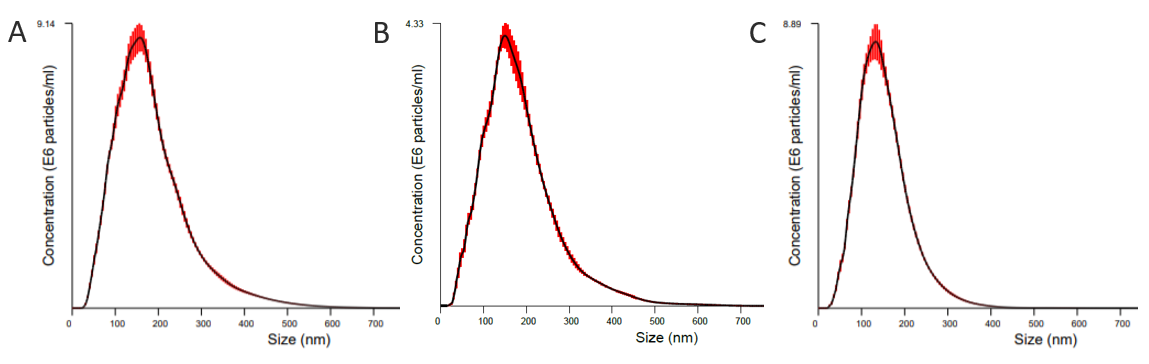
***

***Figure S1.*** *Particle size distribution of* ***(A)*** *rEVs,* ***(B)*** *HEK293 endogenous EVs* *and* ***(C)*** *MCF7 EVs as determined by NanoSight NTA version 2.3 batch analysis report with the mean size distribution of around 185 nm, 184 nm, and 152 nm, respectively.*

*
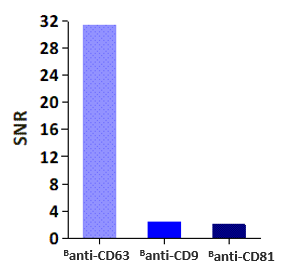
*

***Figure S2.*** *FO-SPR sandwich bioassay with different antibody combinations for detecting spiked MCF7 EVs at 2 x 10^9^ particles/mL concentration, in 100-fold diluted blood plasma. Bar graphs representing the SNR obtained by combining anti-EpCAM capture antibody with different detection antibodies (^B^anti-CD9, ^B^anti-CD63, ^B^anti-CD81).*
